# P111: It is not in your hands!

**DOI:** 10.1186/2047-2994-2-S1-P111

**Published:** 2013-06-20

**Authors:** N Damani, P Yew, S Wallace

**Affiliations:** 1 Infection Prevention and Control, Southern Trust, Craigavon, UK; 2 Infection Prevention and Control, South Eastern Trust, Belfast, UK

## Introduction

Hand hygiene (HH) is well-recognised as part of best practice in preventing cross infection. In light of the *P.aeruginosa* outbreak in neonatal units in the UK, it is recommended that healthcare workers (HCWs) should routinely use alcohol hand rub *after* hand washing in all augmented care areas. Since this practice may lead to dermatitis, we have conducted this study to assess whether use of alcohol hand rub was necessary after hand washing (HW) as we were unable to find any scientific papers to support this recommendation.

## Methods

18 HCWs were recruited for this study. Participants were asked to perform the 6-step technique for HW using soap and water for 40- 60 seconds from *P.aeruginosa* contaminated tap water. After HW, plate impressions were performed from wet fingers and thumbs of both hands onto horse Blood Agar (BA). Participants were then divided equally into two groups; one group was instructed to air dry their hands and another group to dry their hands with disposable paper towels. Impressions from dry fingers and thumbs was taken to BA again from both groups. Following this, all participants were instructed to decontaminate their hands with 3 ml of alcohol hand rub for 20-40 seconds and the process was repeated again for the 3^rd^ time. All BA plates were then incubated at 35^o^C (aerobic) and read at 24 and 48hrs with colony forming unit (cfu) counts performed and recorded.

## Results

At the beginning of the study, 1500 cfu and 5000 cfu per 100ml of *P. aeruginosa* was from hot and cold water taps respectively. At the end of the study, water was resampled for *P. aeruginosa* which showed 0 cfu and 200 cfu per 100ml from hot and cold water taps respectively. No *P.aeruginosa* was isolated in any of the BA plates from 18 HCWs even when the hands were wet and washed with *P.aeruginosa* contaminated tap water. However, significant reduction in the number of all organisms on hands was noticed when the alcohol hand rub was used.

## Conclusion

Use of alcohol hand rub is the method of choice for hand hygiene on physically clean hands. Based in this small study, we can conclude that provided that HW is carried out properly and hands are dried, further antisepsis of hands with alcohol hand rub after HW is not necessary. Further study is needed to confirm our findings.

## Disclosure of interest

None declared

